# Multimodal Strategies to Fight Obesity: Research on Tailored Therapies Based on Natural and Synthetic Compounds for Prevention, Management and Treatment

**DOI:** 10.3390/ijms241210105

**Published:** 2023-06-14

**Authors:** Antonella D’Anneo, Marianna Lauricella

**Affiliations:** 1 Laboratory of Biochemistry, Department of Biological, Chemical and Pharmaceutical Sciences and Technologies (STEBICEF), University of Palermo, 90127 Palermo, Italy; 2 Department of Biomedicine, Neurosciences and Advanced Diagnostics (BIND), Institute of Biochemistry, University of Palermo, 90127 Palermo, Italy

In the past 50 years, the global prevalence of obesity and overweight has tripled, reaching pandemic proportions and blatantly representing an urgent problem for public health [1,2]. Many different factors have actively fed the spread of this chronic condition, from the rising global intake of calorie-dense food to widespread bad behaviors such as living a sedentary lifestyle [3,4]. The most worrying aspect of this condition is its correlation with other comorbidities. Indeed, obesity has been proven to be associated with an increased risk of developing dyslipidemia, hypertension, type 2 diabetes, coronary heart disease, non-alcoholic fatty liver disease (NAFLD), arthritis and even many types of cancers [1,5,6], all of which severely impact the quality and life expectancy of affected subjects.

Thus far, the urgent need for intervention strategies focused on tailored targets, as well as the development of personalized medicine, has been a crucial aim in obesity research [7].

Some of these approaches have been focused on inflammation as a possible target in the development of effective therapeutic strategies [8,9]. Indeed, inflammation in adipose tissues represents a primary force that can contribute to the onset of obesity-associated pathologies [10]. White adipose tissue (WAT) represents an endocrine organ assigned to store lipid deposits and monitor both metabolism and inflammation through the production of adipokines (leptin and adiponectin) and cytokines [11,12]. Indeed, in WAT, many different cell types, such as adipocyte precursors (AP), adipocytes and immune cell subsets (i.e., dendritic cells, T and B cells and macrophages) coexist [13], creating a complicated network for the maintenance of the correct metabolic functionality and integrity of adipocytes. However, substantial evidence has proven that hypoxic conditions fulfill the expansion of adipose tissue and the upregulation of inflammatory response-related adipokines. As a consequence, hypoxia of fat cells increases glucose consumption, promoting the development of adipocyte insulin resistance and adipose tissue fibrosis [14].

In this scenario, a consistent amount of extracellular matrix (ECM) proteins (i.e., fibronectin and many different types of collagen) released by adipocytes, adipocyte progenitors and fibroblasts accumulate in ECM, modifying WAT plasticity and its functionality [15,16], two characteristic events which occur in adipose tissue fibrosis. A pro-fibrotic action in this process is carried out by cytokine secretion by immune system cells as adipose tissue macrophages and mast cells [17]. In a recent study, Arndt et al., using an ex vivo WAT organotypic culture system, identified IL-13 and IL-4 as critical pathogenic mediators of WAT fibrosis. The authors demonstrated that this effect is dependent on WAT-associated macrophages, since their removal by clodronate liposome treatment decreased the fibrotic deposition in WAT in mice intraperitoneally injected with IL-4. A strong positive correlation between fibrosis markers and IL-13/IL-4 receptors was found, but the data seem to indicate that both IL-13 and IL-4 can play a role in ex vivo WAT systems, though only partly in in vivo models [18].

The state of chronic inflammation which characterizes obese subjects contributes to the development of several chronic diseases [11]. Regarding this connection, it has been reported that obesity represents a risk factor for myofascial disease, one of the leading causes of physical disability. Ugwoke et al. highlighted how several pathological processes in obesity, including changes in adipose tissue metabolism, chronic inflammatory state and oxidative stress, may alter the mechanical and biological properties of fascial hyaluronan [19]. Hyaluronan is the main polysaccharide of the ECM of connective tissues; it provides mechanical stability and acts as a water reservoir and lubricant. Moreover, hyaluronan, by binding to cell-surface receptors in adipose tissue, is able to modulate adipogenesis and adipose tissue metabolism. Alterations in the physical and chemical properties of hyaluronan alter the viscoelasticity of the matrix and deregulate molecular signaling, contributing to the development of myofascial disease in obesity.

Some investigations have focused on the identification of pro-inflammatory biomarkers in the saliva of obese subjects. A higher level of some matrix metalloproteinases (i.e., gelatinases MMP-2 and MMP-9), as well as of IL-1β, were found in the saliva of obese women compared to the control group, while in obese men, higher contents of MMP-9, IL-6 and resistin were observed compared to individuals of normal body weight [20].

Zazula et al. demonstrated that an early and sustained inflammatory state favors the acquisition of persistent muscle changes and typical obesogenic features [21]. Such a study was performed via subcutaneous injection of monosodium glutamate (MNG) in Wistar rats. When administered in the perinatal phase, MNG provokes lesions of hypothalamic nuclei in animal models, leading, in adult life, to hyperphagia and unbalanced consumption of nutrients, two typical features of obesity [22,23,24]. In line with this evidence, Zazula’s data demonstrated that MSG exposure promoted adiposity in Wistar rats, favoring a hyperinsulinemic and pro-inflammatory state that were accompanied with fibrosis, oxidative injury and muscle mass reduction in adult rats. The plasmatic profiles of the animals showed remarkable increases in glucose content, total cholesterol, LDL and VLDL, as well as in the amount of triacylglycerols. The analysis of muscle markers in MSG-treated rats provided evidence of an increase in lactate content and a decrease in creatine kinase with respect to the control group [21].

The main cause leading to the accumulation of fat in adipose tissue is an excessive food intake. Several reports have suggested that high sugar consumption is contributing to the global rise in obesity and type 2 diabetes. To reduce sugar intake and its dangerous consequences, the ingestion of polyols, natural sweeteners with low caloric content and a low glycemic index, can be useful [25]. Erythriol and xylitol are two polyols which are partly adsorbed in the small intestine. Bordier et al. evaluated the enteral adsorption of erythriol and xylitol and their potential metabolization into the oxidate form erytronate, whose implications for human health remain to be determined, in healthy volunteers [26]. Based on the results, the authors demonstrated that erythriol is dose-dependently adsorbed and metabolized in small amounts to erytronate, whereas xylitol absorption is low, and no metabolization to erytronate takes place.

Other studies have demonstrated that a high-fat diet (HFD) can contribute to adiposity and obesity status [27]. In particular, the presence of high levels of long-chain saturated fatty acids (FAs), such as palmitate, in the diet has been associated with hypertrophic and dysfunctional adipocytes, as well as with a state of low-grade inflammation in white adipose tissue (WAT) [28]. Thus, reducing the hypertrophy adipose tissue represents a strategy to counteract the detrimental effects of obesity.

Nowadays, numerous foods rich in antioxidants, phytochemicals and essential oils have been found to be helpful in maintaining body weight, and can be considered protective and/or therapeutic against obesity [29,30]. Mango (*Mangifera indica* L.) is a food appreciated for its nutritive and nutraceutical properties. Different parts of the mango plant and fruit have been reported to exert anti-tumoral, antioxidant and anti-inflammatory effects due to the high content of polyphenols [31,32,33]. Pratelli et al. demonstrated that extracts of Sicilian mango peels and seeds, the main bio-wastes of mango processing, are capable of counteracting in 3T3-L1 adipocyte lipotoxicity induced by high doses of palmitate, the main long-chain FA present in the diet. In particular, mango extracts counteracted palmitate-induced hypertrophy by reducing lipid droplets and triglyceride content, as well as reducing endoplasmic reticulum stress induced by palmitate. The lipolytic and antioxidant effects exerted by mango peel and seed extracts seem to be mediated by the activation of the AMPK and Nrf2 antioxidant pathway [34].

A beneficial effect against HFD-induced lipotoxicity also seems to be also exerted by 4-methylesculetin (6,7-dihydroxy-4-methylcoumarin, 4-ME), a coumarin derivative isolated from Artemisia annua [35]. Li et al. showed that 4-ME treatment attenuated adipocyte hypertrophy, macrophage infiltration, hypoxia and fibrosis in epididymal adipose tissue in HFD-fed mice, thus improving the adipose tissue microenvironment. In addition, 4-ME reduced liver fibrosis by lowering FAs uptake and de novo lipogenesis. These effects are correlated with the ability of 4-ME to down-regulate CD36; the free FA cell-surface receptor; as well as SREBP-1, PPAR-γ and FASN protein, transcription factors and enzymes that are involved in lipogenesis. Furthermore, 4-ME activated Nrf2, an important antioxidant transcriptional factor that can also indirectly suppress the expression of SREBP-1 and its lipogenic target genes [35].

A natural compound that has attracted the interest of researchers is curcumin, a polyphenol extracted from the rhizome of *Curcuma longa* L. A consistent piece of evidence has demonstrated that it has different pharmacological properties, including anti-inflammatory, antioxidant, neuroprotective and anti-tumoral effects [36], and it is also able to improve glucose and lipid metabolism [37]. However, the potential therapeutic application of this molecule is strongly limited by its scarce bioavailability as a consequence of its low solubility in water and rapid clearance [38]. Combinatorial treatments aimed at improving both curcumin bioavailability and its half-life have identified piperine, an alkaloid extracted by *Piper nigrum* L. and *Piper longum*, as a possible candidate to be co-administered with curcumin. However, in a recent case study, Servida et al. demonstrated that this combination treatment should be better explored. Indeed, in a patient exposed to a low-altitude condition, curcumin/piperine co-administration induced severe hypoglycemia followed by a transient loss of consciousness [38].

Several studies support the conclusion that obesity represents a risk factor for the development of neurodegenerative diseases, including dementia and Alzheimer’s disease [39]. Indeed, obesity is associated with chronic low-grade inflammation and oxidative stress, which contribute to the onset and progression of neurodegeneration. There is an assumption that foods rich in antioxidants may exert protective effects on neurodegeneration. To this end, Terzo et al. evaluated the effect of combined administration of Sicilian black bee chestnut honey and D-limonene, which are known to mitigate inflammation and oxidative stress, in HFD-fed mice. After 10 weeks of consuming an HFD, the mice developed neuronal apoptosis, increased pro-inflammatory cytokines and oxidative stress markers. Interestingly, all of these alterations were counteracted by the combined administration of honey and limonene [40]. Notably, they also reduced amyloid plaque processing and improved synaptic function, thus suggesting that a honey and limonene combination can represent a potential dietary supplement to counteract HFD-induced brain damage.

In addition to natural compounds, some synthetic molecules with targeted action showed promising effects for reducing obesity and related diseases. Adenosine receptor subtypes A2A and A2B represent important therapeutic targets for the treatment of obesity [41]. Theophylline is a non-selective adenosine receptor antagonist that has been shown to reduce body weight in obese animals [42]. However, its side effects, such as hyperactivity and heart rhythm disturbances, represent a problem. Kotańska et al. [43] compared the effect of theophylline with that of PSB-603, a specific adenosine A2B receptor antagonist, on high-fat/high-sugar diet-fed mice and demonstrated that both the A2B receptor antagonists significantly lowered the body weights of the mice. However, only PSB-603 was also capable of reducing triglycerides and total cholesterol blood levels in mice, thus suggesting that blocking A2A with a specific antagonist has stronger effects on lipid reduction than non-selective inhibition.

In addition, several studies supported the existence of an anti-obesity effect of compounds stimulating histamine release [44]. The histaminergic system is involved in the regulation of food intake and body weight control [44]. Stimulation of the H3 receptor activates a negative feedback due to histamine release, while H3 receptor inhibition by specific antagonists shows efficacy in inhibiting weight gain [45]. In a recent publication, Mika et al. demonstrated that KSK-74, a new specific H3R antagonist, reduced weight gain in overfeeding rats. It also improved their glucose tolerance and adipocyte hypertrophy, suggesting its potential use as an anti-obesity compound [46].

Beyond these emerging data, new insights for the development of therapeutic strategies against obesity come from the identification of epigenetic molecular targets. During adipogenesis, a highly orchestrated gene expression program occurs [47]. It favors the cell commitment of pluripotent stem cells (first stage) into pre-adipocytes, and then the terminal differentiation of pre-adipocytes into mature adipocytes (second stage) [48]. A complex system of epigenetic changes and chromatin remodeling processes alternates during the differentiation flux, causing the silencing of stemness-associated genes [49]. In recent years, such an aspect has been a hot topic in the scientific community, pushing the search for natural and synthetic compounds able to modulate that intricate network of proteins and transcription factors that are directly involved in stem cell differentiation or in uncontrolled adipocyte tissue hyperproliferation [50,51]. In this context, metformin and vitamin D have been revealed as very promising therapeutic agents, since their administration to adipose-derived stem cells upregulates HDAC1 expression, affected the stem cell phenotype and modulated the expression of those miRNAs playing a key role in stem cell adipogenesis [47,52].
